# Miliary tuberculosis with co-existing pulmonary cryptococcosis in non-HIV patient without underlying diseases: a case report

**DOI:** 10.1186/s12890-018-0578-8

**Published:** 2018-01-16

**Authors:** Toyomitsu Sawai, Takumi Nakao, Satoru Koga, Shotaro Ide, Sumako Yoshioka, Nobuko Matsuo, Hiroshi Mukae

**Affiliations:** 1 Department of Respiratory Medicine, Nagasaki Harbor Medical Center, 6-39 Shinchi-machi, Nagasaki, 850-8555 Japan; 2 Second Department of Internal Medicine, Nagasaki University Hospital, 1-7-1 Sakamoto-machi, Nagasaki, Japan

**Keywords:** Miliary tuberculosis, Pulmonary cryptococcosis, Co-infection, Non-HIV patient without underlying diseases

## Abstract

**Background:**

Tuberculosis and cryptococcosis co-infection usually occurs in immunosuppressed patients with impaired cell-mediated immunity. However, there are few reports about such co-infection in non-HIV patients without underlying diseases. Here, we report a case of miliary tuberculosis with co-existing pulmonary cryptococcosis in non-HIV patient without underlying diseases.

**Case presentation:**

An 84-year-old Asian female presented to our hospital with complaints of a 1-week history of abdominal pain and appetite loss. Chest computed tomography (CT) showed diffuse micronodules in random patterns in both lung fields. Liver, skin and bone marrow biopsies showed epithelioid cell granuloma. Polymerase chain reaction of gastric aspirate was positive for *Mycobacterium tuberculosis*. According to these findings, miliary tuberculosis was suspected and antimycobacterial therapy was initiated. After a 6-month treatment course, chest radiograph showed new multiple nodules in the right middle lung field. Chest CT showed that a right S6 small nodule was increased and new multiple nodules appeared in the right lower lobe. Flexible fiberoptic bronchoscopy was subsequently perfomed. Cytology of the bronchial lavage showed a small number of Periodic acid-Schiff-positive bodies, suggesting *Cryptococcus* species. Moreover, serum cryptococcal antigen testing was positive. According to these findings, pulmonary cryptococcosis was diagnosed, although the culture was negative. Oral fluconazole therapy was subsequently initiated. After a 6-month treatment course, chest radiograph showed gradual improvement.

**Conclusion:**

Although tuberculosis and cryptococcosis co-infection is relatively rare in immunocompromised hosts, such as those with acquired immunodeficiency syndrome, clinicians should be aware that these infections can co-exist even in non-HIV patients without underlying diseases.

## Background

There are an increasing number of cases of either tuberculosis or cryptococcosis due to impaired cellular immunity in immunocompromised patients such as those with acquired immunodeficiency syndrome (AIDS) and diabetes mellitus or receiving corticosteroids or immunosuppressive agents [1]. However, tuberculosis and cryptococcosis co-infection is seldom reported even in immunocompromised patients. Particularly, co-infection of these diseases is extremely rare in immunocompetent patients. Here, we present a case of miliary tuberculosis with co-existing pulmonary cryptococcosis in non-HIV patient without underlying diseases.

## Case presentation

**Fig. 1:**
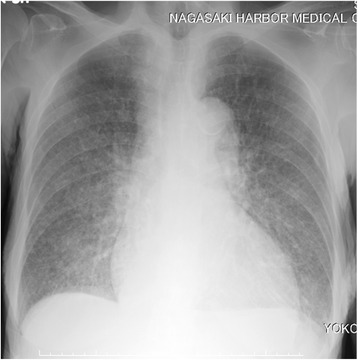
Chest radiography on admission showed diffuse micronodules in both lung field

**Fig. 2:**
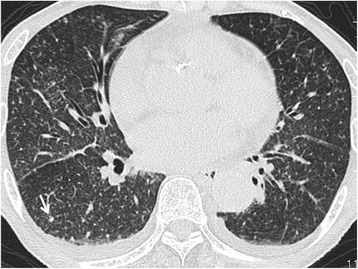
Chest CT on admission showed diffuse micronodules at random pattern in both lung field and a small nodule in the right S6 (white arrow)

**Fig. 3:**
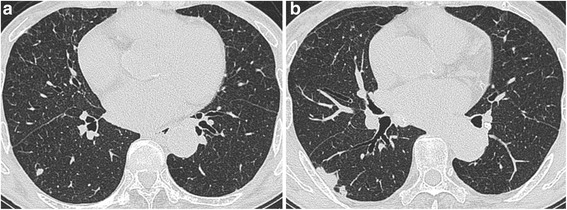
Chest CT showed that a right S6 small nodule was increased (**a**) and new multiple nodules appeared in the right lower lobe (**b**)

**Fig. 4:**
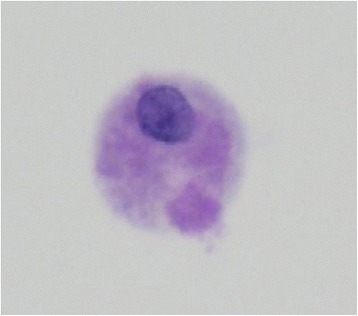
Cytology of the bronchial lavage showed small amount of body suspicious for *Cryptococcus* species (Periodic acid-Schiff stain, ×400)

An 84-year-old woman presented to our hospital with complaints of a 1-week history of abdominal pain and appetite loss. The patient had no history of cough, sputum, fever, chills, weight loss, or night sweats. She had no history of tobacco smoking, tuberculosis or exposure to individuals with tuberculosis. She did not have a history of malignancy, diabetes mellitus, cytotoxic therapy or corticosteroid use. Her family history was unremarkable. Physical examination revealed a heart rate of 90 beats/min, blood pressure of 157/76 mmHg, respiratory rate of 24 breaths/min, temperature of 37.8 °C and oxygen saturation of 98% on room air. Respiratory, cardiac and abdominal examination were unremarkable. Chest radiograph showed multiple small nodules in both lung fields and chest computed tomography (CT) showed diffuse micronodules in random patterns in both lung lobes, cardiomegaly and bilateral pleural effusion (Figs. 1 and 2). White blood cell count, C-reactive protein and procalcitonin levels were 2800 /μl, 7.50 mg/dl (normal range 0.00–0.10 mg/dl) and 0.373 ng/ml (normal range 0.000–0.046 ng/ml), respectively. Serum carcinoembryonic antigen, carbohydrate antigen 19–9 and soluble IL-2 receptor were elevated at 9.4 ng/ml (normal range 0.0–5.0 ng/ml), 188.4 U/ml (normal range 0.0–37.0 U/ml) and 6163 U/ml (normal range 0–500 U/ml), respectively. Angiotensin-converting enzyme, mycoplasma antibody and blood sugar were within normal ranges. Serum QuantiFERON testing was positive. CD4/8 ratio, CD4 count and CD8 count were 1.73 (normal range 0.6–2.9), 422 /μl (normal range 344–1289) and 244 /μl (normal range 110–1066), respectively. IgG, IgA and IgM were 998 mg/dl (normal range 870–1700), 128 mg/dl (normal range 110–410), 59 mg/dl (normal range 46–260), respectively. Testing for human immunodeficiency virus infection was negative. Expectorated sputum smears were negative for bacteria and acid-fast bacilli. Urinary antigen testing (Binax NOW; Binax, Inc., Portland, ME) for *Streptococcus pneumoniae* and *Legionella pneumophila* was negative. According to these results, we initially suspected intraabdominal malignancy including malignant lymphoma. However, abdominal CT and magnetic resonance imaging showed no abnormalities. Therefore, we suspected miliary tuberculosis or pulmonary sarcoidosis. Liver, skin and bone marrow biopsies were subsequently performed and showed epithelioid cell granuloma without caseous necrosis. Gastric aspirate smear was positive for acid-fast bacilli and polymerase chain reaction (Loopamp; Eiken Chemical Co., Ltd. Tokyo, Japan) was positive for *Mycobacterium tuberculosis*. Although these microbiological findings might indicate the presence of non-viable *M. tuberculosis*, miliary tuberculosis was suspected and antimycobacterial therapy [oral isoniazid (INH) 200 mg/day, rifampicin (RFP) 300 mg/day and ethambutol (EB) 500 mg/day] was initiated on hospital day 12. Gastric aspirate culture was positive for *M. tuberculosis* after 1 week of culture. After a 2-month treatment course, chest radiograph showed gradual improvement, oral EB was discontinued and the patient was discharged. Although INH and RFP therapy was continued, chest radiograph showed new multiple nodules in the right middle lung field after a 6-month treatment course. Chest CT showed that a right S6 small nodule, presumed to be miliary tuberculosis, had increased and new multiple nodules appeared in the right lower lobe (Fig. 3). The patient’s white blood cell count and C-reactive protein at this time were 2400 /μl and 0.09 mg/dl, respectively. Flexible fiberoptic bronchoscopy was subsequently perfomed. Microbiological testing of bronchial lavage fluids did not reveal any bacteria, mycobacteria or fungi. However, cytology showed a small number of Periodic acid-Schiff-positive bodies, suggesting *Cryptococcus* species (Fig. 4). Moreover, serum cryptococcal antigen testing (Serodirect “EIKEN” Cryptococcus; Eiken Chemical Co., Ltd. Tokyo, Japan) was positive (×128). According to these findings, pulmonary cryptococcosis was diagnosed, although the culture was negative. Oral fluconazole (FLCZ; 300 mg/day) was subsequently initiated. After a 6-month treatment course, chest radiograph showed gradual improvement and oral FLCZ was discontinued. The patient received a total of 12 months of antimycobacterial therapy. On follow-up, she has remained asymptomatic with suspect to pulmonary disease, with no recurrence.

## Discussion and conclusions

Both tuberculosis and cryptococcosis have a wide range of clinical presentations, varying from pulmonary infection to the systemic infection. These diseases are more common in patients with impaired cell-mediated immunity such as those with AIDS, hemodialysis, hematologic malignancies, cancer and diabetes mellitus or receiving corticosteroids or immunosuppressive agents [1, 2]. Especially, this co-infection is almost always indicative of compromised cell-mediated immunity. Thus, its occurrence is extremely rare in immunocompetent patients. The first report of concomitant tuberculosis and cryptococcosis in immunocompetent patients was reported in 1966 [3]. Since that initial report, several cases of concurrent infection of tuberculosis and cryptococcosis in immunocompetent patients have been reported [3–12] (Table 1). Most reported cases with tuberculosis and cryptococcosis co-infection involved the lung and central nervous system, respectively. Aydemir H et al. reported a case of *C. neoformans* meningitis in an HIV-negative patient suspected of having miliary tuberculosis [13]. However, the authors did not definitively diagnose the patients with miliary tuberculosis. To our knowledge, a case of miliary tuberculosis with co-existing pulmonary cryptococcosis, even in immunocompromised patients, has not been reported previously. Additionally, despite an extensive evaluation, we found no evidence of immunodeficiency in our patient. In conclusion, the described patient was diagnosed with military tuberculosis with a co-existing pulmonary cryptococcal infection in non-HIV patient without underlying diseases.

**Table 1:** Reported case of co-infection tuberculosis and cryptococcosis in non-HIV patient without underlying diseases

Case/Ref	Age/sex	Region	Pathological lesions (tuberculosis/cryptococcosis)	Treatment (tuberculosis/cryptococcosis)	Outcome
1/3)	61/M	United States	Lung/CSF	INH, SM/AMPH-B	Recovered
2/4)	69/M	United States	Lung/Lung	INH, RFP/KCZ	Recovered
3/5)	51/M	Spain	CSF/CSF	INH, RFP, EB, PZA/AMPH-B, 5-FC	Recovered
4/6)	34/F	Saudi Arabia	Lymph node/Vertebra	INH, RFP, EB, PZA/FLCZ	Recovered
5/7)	25/F	Italy	CSF/CSF	INH, RFP, EB, PZA, SM/FLCZ, L-AMB	Recovered
6/8)	18/F	Canada	Lung/CSF, Lymph node	INH, RFP, EB, PZA/AMPH-B, 5-FC, FLCZ	Recovered
7/9)	65/M	India	Lung/Lung	NA/AMPH-B, ITCZ	Recovered
8/10)	58/F	Taiwan	Lymph node/Lung	NA/FLCZ	Recovered
9/11)	70/M	Iran	Lung/CSF, Lung	INH, RFP, EB, PZA/AMPH-B	Died
10/12)	61/M	Rwanda	Lung, Bone marrow, Liver/CSF	INH, RFP, EB/AMPH-B, FLCZ	Recovered
11/present case	84/F	Japan	Lung, Bone marrow, Liver, Skin/Lung	INH, RFP, EB/FLCZ	Recovered

*NA* Not available, *CSF* Cerebrospinal fluid, *INH* Isoniazid, *SM* Streptomycin, *AMPH-B* Amphotericin B, *RFP* Rifampicin, *KCZ* Ketoconazole, *EB* Ethambutol, *PZA* Pyrazinamide, *5-FC* 5-fluorocytosine, *FLCZ* Fluconazole, *L-AMB* Liposomal amphotericin B, *ITCZ* Itraconazole, *HIV* Human immunodeficiency virus

In this case, CT images on the day of admission showed diffuse micronodules in random patterns in both lung fields and a small nodule in the right S6. Although both miliary tuberculosis and disseminated cryptococcosis present diffuse micronodules in random patterns in chest CT images, diffuse micronodules decreased following antimycobacterial treatment. Accordingly, these images were compatible with a diagnosis of miliary tuberculosis. However, the small nodule in the right S6 increased in size despite antimycobacterial therapy but reduced in size after antifungal treatment. Therefore, the small nodule was considered to be a primary focus of pulmonary cryptococcosis. The radiological findings of pulmonary cryptococcosis are well known. The common CT findings are nodular lesions, alveolar infiltrates, ground glass attenuation, cavitation, linear opacities, septal thickening, pleural effusion and lymphadenopathy [14]. In our case, the diagnosis of cryptococcosis was delayed because we believe that the small nodule was suggestive of miliary tuberculosis or post-inflammatory change. When pulmonary cryptococcosis appears as a small nodule combined with miliary tuberculosis, it might be very difficult to distinguish between these two diseases. We usually consider abnormal shadows to have the same etiology. However, pulmonary tuberculosis and pulmonary cryptococcosis are known to exhibit non-specific CT findings, such as alveolar infiltrates, nodules, micronodules, lymph node swelling and pleural effusion, and there are no characteristic findings [15]. Therefore, helpful diagnostic tools such as QuantiFERON test and serum cryptococcal antigen test should be considered. However, Dotsu et al. reported that sensitivity of the serum cryptococcal antigen test was decreased when the nodule size measured <15 mm [16]. Although we did not examine serum cryptococcal antigen on admission, its result would likely have been negative due to the small nodule size in this case.

Although impaired cellular-mediated immunity is a known risk factor for both mycobacterial and cryptococcal infections, the results of CD4 and CD8 counts and immunoglobulins in the present case were normal. Previous studies have demonstrated that tuberculosis causes alternations in cellular immunity and is recognized as a predisposing factor for developing cryptococcosis [17, 18]. However, high melanin-producing strains of *C. neoformans* inhibit T cell-mediated immunity such as the production of tumor necrosis factor-alpha and lymphoproliferation, thereby predisposing patients to tuberculosis reactivation or infection [19]. Thus, there is some evidence that both infections have immunomodulatory effects on host defenses. However, it is difficult to determine whether tuberculosis preceded cryptococcosis or vice versa in this case.

Because miliary tuberculosis with co-existing pulmonary cryptococcosis is not common, its presence may easily be overlooked, especially, if the nodule is very small. The present case emphasizes the fact that radiological findings of the two infections may be confusing when both co-exist in the lungs. Clinicians should take into consideration that these infections can co-exist even in immunocompetent patients.

In conclusion, we described a case of miliary tuberculosis with co-existing pulmonary cryptococcosis in non-HIV patient without underlying diseases. Although rare, clinicians should be aware of the possibility that these infections can co-exist even in immunocompetent patients.

## Abbreviations

EB: Ethambutol
FLCZ: Fluconazole
INH: Isoniazid
RFP: Rifampicin
